# In Vitro Anticancer Drug Sensitivity Sensing through Single-Cell Raman Spectroscopy

**DOI:** 10.3390/bios11080286

**Published:** 2021-08-20

**Authors:** Jingkai Wang, Kaicheng Lin, Huijie Hu, Xingwang Qie, Wei E. Huang, Zhisong Cui, Yan Gong, Yizhi Song

**Affiliations:** 1 Division of Life Sciences and Medicine, School of Biomedical Engineering (Suzhou), University of Science and Technology of China, Hefei 230026, China; wangjk@sibet.ac.cn (J.W.); huhj@sibet.ac.cn (H.H.); 2 Suzhou Institute of Biomedical Engineering and Technology, Chinese Academy of Sciences, Suzhou 215163, China; linkc@sibet.ac.cn (K.L.); qiexw@sibet.ac.cn (X.Q.); 3 Department of Engineering Science, University of Oxford, Parks Road, Oxford OX1 3PJ, UK; wei.huang@eng.ox.ac.uk; 4 Marine Bioresources and Environment Research Center, First Institute of Oceanography, Ministry of Natural Resources of China, Qingdao 266061, China; 5 Laboratory for Marine Ecology and Environmental Science, Qingdao National Laboratory for Marine Science and Technology, Qingdao 266071, China

**Keywords:** chemotherapy, drug efficacy, cancer cell, Raman spectroscopy, single cell

## Abstract

Traditional in vitro anticancer drug sensitivity testing at the population level suffers from lengthy procedures and high false positive rates. To overcome these defects, we built a confocal Raman microscopy sensing system and proposed a single-cell approach via Raman-deuterium isotope probing (Raman-DIP) as a rapid and reliable in vitro drug efficacy evaluation method. Raman-DIP detected the incorporation of deuterium into the cell, which correlated with the metabolic activity of the cell. The human non-small cell lung cancer cell line HCC827 and human breast cancer cell line MCF-7 were tested against eight different anticancer drugs. The metabolic activity of cancer cells could be detected as early as 12 h, independent of cell growth. Incubation of cells in 30% heavy water (D_2_O) did not show any negative effect on cell viability. Compared with traditional methods, Raman-DIP could accurately determine the drug effect, meanwhile, it could reduce the testing period from 72–144 h to 48 h. Moreover, the heterogeneity of cells responding to anticancer drugs was observed at the single-cell level. This proof-of-concept study demonstrated the potential of Raman-DIP to be a reliable tool for cancer drug discovery and drug susceptibility testing.

## 1. Introduction

The discovery, screening, and administration of safe and effective anticancer drugs are vital for tackling cancers. In vitro drug testing is a key step in evaluating the efficacy of anticancer drugs. It is extensively carried out not only during drug development to screen candidates entering clinical trials but also before medical treatment to find the right medication for individual patients when many drugs become ineffective due to drug resistance. Considering the long duration and high cost of drug development, as well as the consequences of inappropriate medication, it is important to develop reliable and effective platforms for screening anticancer molecules [1,2].

In vitro drug screening is traditionally performed on cell lines by examining their viability or proliferation after exposure to drugs. These methods detect the cell viability at the population level by measuring cellular oxidoreductase (e.g., NADP(H) and dehydrogenase) activity or adenosine triphosphate (ATP) synthesized only in viable cells. Common methods of in vitro screening include a colorimetric tetrazolium reduction (MTT) assay or other commercially available derivatives such as cell counting kit-8 (CCK-8) or CellTiter-Glo kit (CTG) [3].

However, traditional in vitro drug sensitivity testing methods have several limitations that contribute to the high drug attrition rates in oncology and incorrect susceptibility results for precision medication [4]: (1) Cell viability tests often have false results. For example, reagent-associated toxicity issues contribute to false positives [5]. In addition, the redox potential, oxidative stress, and other oxidoreductases that react with the colorimetric substrate also affect the results [6]. (2) Traditional assays normally require a long duration (72–144 h), during which time alterations in the media, drugs, and cells can impact the results [7]. Long procedural delays can prevent patients from receiving appropriate treatment. (3) The heterogeneity of tumor cells plays an important role in cancer development, as well as cancer drug resistance. Unfortunately, population-based experiments fail to detect variations in the cell population and intra-sample heterogeneity [8]. Hence, the development of in vitro anticancer drug sensitivity detection which can involve less toxic or non-toxic compounds, shorten the procedure and reflect single-cell heterogeneity is critical to overcome these issues.

Raman scattering is commonly used in diagnosis because it is a fast, label-free, non-invasive and molecular-specific technology [9]. Surface-enhanced Raman-scattering (SERS) has been used in evaluating anticancer-drug efficacy [10,11]. However, universal SERS biomarkers for predicting anticancer-drug effects have not been reported. Single-cell Raman spectra (SCRS) detect the vibrational modes of biomolecules in a cell and reflect the biochemical profile or phenotype at the single-cell level. Cells/bacteria can be labeled with a stable isotope, such as ^13^C, ^15^N, and ^2^H(D), and exhibit characteristic Raman spectra shift due to the replacement of heavy stable isotopic atoms in biomolecules [12,13]. We previously proposed a Raman-deuterium isotope probing (Raman-DIP) approach to determine the antibiotic-resistant bacteria in environmental [14] and clinical samples [15] by coupling deuterium labeling and single-cell Raman spectroscopy. The rationale behind this approach is that only metabolically active cells in the presence of antimicrobial agents and D_2_O could integrate deuterium and add D into biomolecules as carbon–deuterium (C–D) bonds. This leads to a characteristic Raman C–D band at 2000–2300 cm^−1^ [14]. The C–D Raman band is a universal biomarker for metabolic activity identification of various types of bacteria and archaea [14,16,17,18,19,20]; however, studies that address the feasibility of applying Raman-DIP to reflect the metabolic activity of cancer cells and to correlate single-cell metabolic activity with anticancer drug screening are rare.

The aim of this study was to propose and evaluate a new reliable and effective in vitro anticancer drug sensitivity approach, based on the detection of cell line metabolic activity via Raman spectroscopy at the single-cell level. The concept of this approach was demonstrated in two cancer cell lines (HCC827 and MCF-7) and eight anticancer drugs. The metabolic activity of cancer cells could be detected as early as 12 h, independent of cell growth. We then verified its feasibility by comparing it with classical in vitro drug sensitivity tests. The new sensing approach involves no toxic chemicals and reduces the test time to 48 h. More importantly, single-cell Raman-DIP sensing can analyze single-cell heterogeneous responses to chemotherapy drugs, which cannot be achieved with conventional approaches.

## 2. Materials and Methods

### 2.1. The Setup of a Raman Microscopy Sensing System

**Figure 1:**
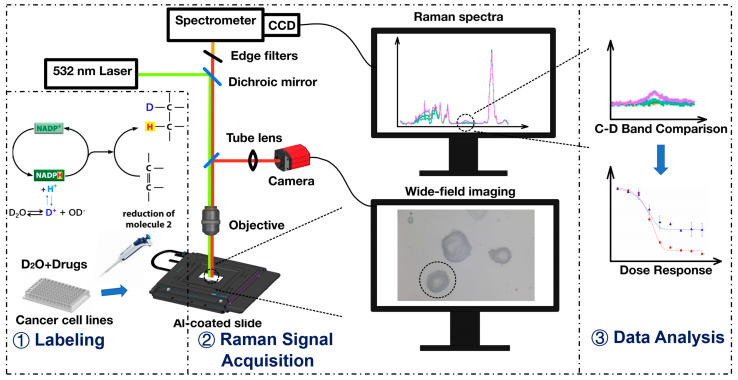
Schematic of the home-built Raman microscopy system and the experiment steps.

A schematic of the home-built Raman microscopy system is shown in Figure 1. The system contained a continuous laser (DPL 532 nm 100 mW, Cobolt, Solna, Sweden), a home-built upright confocal Raman microscopy module adapted from Olympus upright microscope (BX43F, Olympus, Tokyo, Japan), a motorized x-y-z stage (H1P4BX, Prior, Fulbourn, Cambridge, UK), a home-built spectrometer with a CCD (charge-coupled device) (iXon Ultra 888 Ultra EMCCD, Andor, Abingdon, Oxon, UK), and a wide-field imaging camera (Kiralux CS895CU, Thorlabs, Newton, NJ, USA). Detailed system information is listed and shown in Figure S1 and Table S1. The 532 nm laser was used to excite the Raman scattering of single cells. The motion stage was equipped with a slide holder to hold sample slides and was controlled at 0.1 μm accuracy. The confocal microscope was used to collect Raman scattering signal and bright-field images. The Raman scattering signal was recorded with the CCD. The system can provide a spectral resolution of 3 cm^−1^ and a span of 400–3400 cm^−1^ wavenumber range by a grating (600 lines/mm).

### 2.2. Cell Culture

This work used the human non-small cell lung cancer cell line HCC827 and human breast cancer cell line MCF-7 (Mlbio, Shanghai, China). The cells were cultured in a mixture of 90% RPMI 1640 (Sigma Aldrich, Shanghai, China, 10% FBS serum (ATTC, Manassas, VA, USA) and 1% penicillin/streptomycin (Invitrogen, Carlsbad, CA, USA). The cells were grown in an incubator at 37 °C with 5% CO_2_. The cells were then sub-cultured every three days to maintain at least 50% confluence.

### 2.3. Deuterium Labeling and Raman Fingerprint Acquisition

MCF-7 and HCC827 cells (approximately 1 × 10^4^ cells/well) were seeded in 96-well plates and grown overnight. The media were then removed and replaced with RPMI 1640 containing 0%, 5%, 10%, 20%, 30%, and 40% D_2_O (99.9 atom% D, Sigma-Aldrich, St. Louis, MO, USA) for 24 h, respectively. Three replicates were performed for each D_2_O concentration. After 24 h, cells were removed from the wells with 0.25% trypsin (Macgene, Beijing, China) and were fixed with 4% paraformaldehyde for 10 min to maintain their original morphology and avoid cytolysis. To investigate the influence of labeling time, cells were incubated in RPMI 1640 containing 30% D_2_O for 48 h in 15 replicating wells. Every 12 h, cells were removed from 3 of the 15 wells and fixed. The fixed cells were then washed with deionized water three times to remove the medium. Next, 2 μL of the cells was transferred to an aluminum-coated slide (ThermoFisher, Shanghai, China); because they provide relatively low background Raman signals, a higher signal-to-noise ratio could be guaranteed. Then, the cells were air-dried in a laminar flow chamber together with the slides. The spontaneous Raman spectra were excited with 7 mW laser power at the samples; an integration time of 2 s was used per spectrum. We randomly picked at least 10 cells in each replicate and collected 10 Raman spectra for each cell at 10 randomly selected positions.

### 2.4. Toxicity of Deuterium to Cells

MCF-7 and HCC827 cells (approximately 1 × 10^4^ cells/well) were seeded in 96-well plates and grown overnight. The media were then removed and replaced with RPMI 1640 containing 0%, 10%, 20%, 30%, and 40% D_2_O for 24 h, with eight replicate wells for each D_2_O concentration. The cell viability was monitored after 24 h via a CCK-8 assay, following the supplier’s instructions (Vitascientific, Beltsville, MD, USA). The cells cultured in the wells contained RPMI 1640 with 0%, 10%, 20%, 30%, and 40% H_2_O serving as controls.

### 2.5. Anticancer Drug Treatment of Cells

The eight anticancer drugs used in this study were non-targeted drugs (cisplatin, gemcitabine, and monomethyl auristatin E (MMAE)) and targeted drugs (osimertinib, afatinib, crizotinib, gefitinib, and icotinib); these were from Sigma-Aldrich. The 10 mM stock solutions of the drugs were prepared by dissolving chemicals in dimethyl sulfoxide (DMSO). The working solutions were prepared by diluting the stock solution 100-fold, followed by serial 3-fold dilutions in phosphate-buffered saline (PBS). After the cells reached 50% confluence, 10 μL of working solution was added to 90 μL of cell cultures in 96-well plates with three replicates for each drug concentration. Two plates with cell culture reagents were treated simultaneously: one set was examined with CTG assay, and the other set used SCRS.

### 2.6. Anticancer Drug Sensitivity Testing via SCRS

After 24 h of exposure to anticancer drugs, D_2_O was added to plates at a final concentration of 30%. Cells were maintained in the plates for another 24 h until 0.25% trypsin (Macgene, China) was added to remove cells. Then, the cells were then fixed, washed, and pipetted to acquire their SCRS following the same experimental procedures described in Section 2.3. At least 100 spectra were acquired in each replicate with an average of 10 spectra per cell. For heterogeneity analysis, an extra 150 spectra were collected from 150 HCC827 cells treated with each concentration of crizotinib.

### 2.7. Anticancer Drug Sensitivity Test by CTG Assay

MCF-7 cells were treated with cisplatin and gemcitabine for 72 h and with MMAE for 144 h. HCC827 cells were treated with gefitinib, cisplatin, afatinib, osimertinib, and icotinib for 72 h, as well as with crizotinib for 144 h. Cell viability was tested with the CTG assay (Promega, Beijing, China) at the end of the treatments. Briefly, CTG reagents were thawed and kept at ambient temperature for 30 min. Reagents (100 μL) were then added to each well of 96-well plates containing 100 μL of cell line culture. The cells were lysed by shaking on a 96-well plate shaker at 500 rpm for 5 min. The plates were then kept on the bench for 20 min to stabilize the luminescence. The intensity of the luminescence was recorded using a Synergy HT plate reader (Bio-Tek, Winooski, VT, USA). Wells without the addition of anticancer drugs served as negative controls.

### 2.8. Data Analysis

Unless otherwise specified, data analysis was performed using scripts developed in the programming language R (Version 4.0.3, R Core Team, 2020), and figures were produced using the package ggplot2 (Wickham, 2016). The Raman spectra were noise-reduced and baseline-corrected in Labspec6 (Horiba JY, Tokyo, Japan) with 10-degree linear baseline fitting algorithm. The spectra were then normalized to the intensity of the phenylalanine ring breathing peak at 1003 cm^−1^, for the peaks that are normally sharp and stable [21]. The extent of deuterium incorporation was shown by a metabolic index, i.e., the percentage of the integrated spectral intensity of the C–D band (2000–2300 cm^−1^) compared with the sum of the C–D band and the C–H band (2800–3100 cm^−1^). The relative cell viability was calculated as the percentage of luminescence from the CTG protocol of the drug-treated sample to that of the negative control sample. The metabolic ratio was defined as the metabolic index of drug-treated samples to the negative control. The dose-effect curve fitting was achieved in Origin 9 software (OriginLab Corporation, Northampton, MA, USA) with a sigmoidal model. Finally, the concentrations that caused a 20%, 30%, 40%, and 50% inhibition in cell activity and metabolic ratio were denoted as IC_20_, IC_30_, IC_40_, and IC_50_, respectively (Table S3). Sensitivity, specificity, and accuracy of each criterion were calculated as described in Supplementary Document (Table S4).

## 3. Results and Discussion

### 3.1. Raman Spectra of Deuterium-Labeled Cancer Single Cells

**Figure 2:**
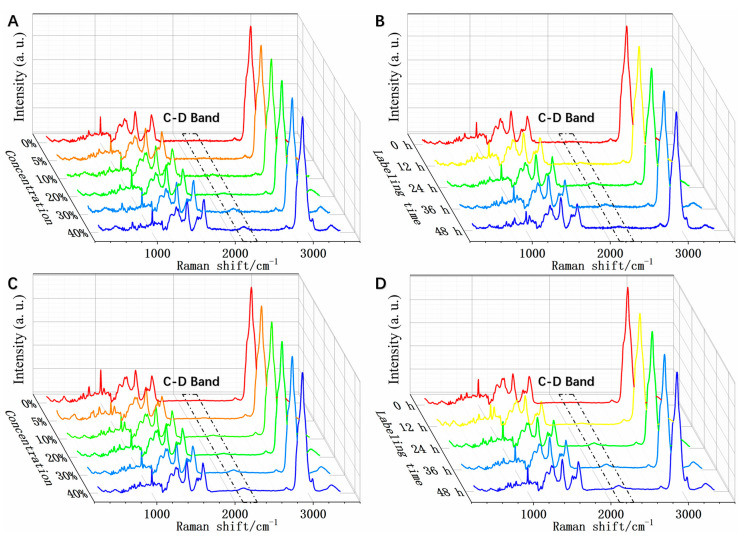
Single-cell Raman Spectra (SCRS) of cells after being incubated in D_2_O-containing medium. (**A**) HCC827 cells incubated with various D_2_O concentrations for 24 h. (**B**) HCC827 cells incubated in 30% D_2_O for different labeling time. (**C**) MCF-7 cells incubated at various D_2_O concentrations for 24 h. (**D**) MCF-7 cells incubated in 30% D_2_O for different labeling time. The lines represent the average intensity (*n* ≥ 100). The spectra were baseline corrected and normalized with the intensity of the phenylalanine peak at 1003 cm^−1^.

To establish the method for deuterium labeling and SCRS acquisition of cancer cell lines, HCC827 and MCF-7 cells were incubated in media containing D_2_O, and their SCRS were analyzed. Figure 2 shows the average SCRS of HCC827 and MCF-7 single cells after being incubated in media containing different concentrations of D_2_O for various times. Compared with the treatments with 0% D_2_O, the SCRS of cells incubated in the D_2_O containing medium with a deuterium concentration as low as 5% displayed a unique Raman band near 2170 cm^−1^ (Figure 2A,C). In contrast, the addition of D_2_O did not change the intensity of Raman peaks for some important biological molecules, such as 781, 1240, and 1450 cm^−1^, that are assigned to nucleic acid, protein, and protein and lipids, respectively (Figure S2). In general, the intensity of the 2170 cm^−1^ band increased as the D_2_O concentration in the medium increased. Figure 2B,D show that the Raman band at 2170 cm^−1^ gradually rose with time when incubated in 30% deuterium. Twelve hours of incubation in D_2_O was enough for the labeling and detection of that band. 

The Raman spectra of biological samples reflect their fingerprints [22,23]; previous studies in which bacteria were cultured in medium containing deuterium suggested that the Raman band at 2170 cm^−1^ was due to the carbon–deuterium (C–D) bond in the newly synthesized lipids and protein [16]. The generation of biological building blocks containing a C–D bond was due to the incorporation of deuterium from D_2_O into biomolecules via NADPH regeneration by metabolically active cells; NADPH regeneration is an intracellular anabolic biochemical process (illustrated in Figure S3). Therefore, there was a shift in the Raman band from 2900 cm^−1^ (carbon-hydrogen bond) to 2170 cm^−1^ (C–D bond) [14,15,24]. The characteristic C–D band emerges in a range known as the ‘silent zone’ (1800–2900 cm^−1^) that usually does not involve vibrational modes contributed by biomolecules formed of naturally occurring isotopes. This is a distinct and easy-to-detect biomarker to evaluate the metabolic activity of a single cell [15]. Figure 2 shows that the eukaryotic cells might undergo a similar deuterium labeling process as prokaryotic cells; the C–D band in the Raman spectra could be a universal metabolic biomarker for cancer cells.

### 3.2. Influence of Deuterium Concentration and Incubating Duration on Deuterium Labeling

**Figure 3:**
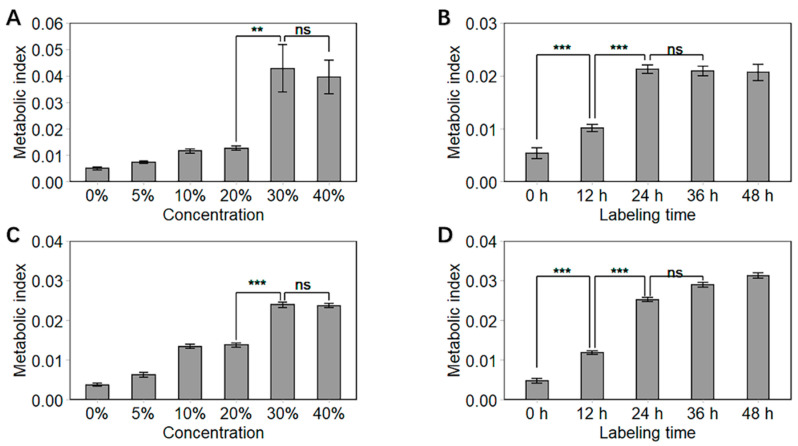
Single-cell metabolic index of HCC827 (**A**,**B**) and MCF-7 (**C**,**D**). Cells were incubated at various D_2_O concentrations for 24 h (**A**,**C**) and in 30% D_2_O for different durations (**B**,**D**). Statistical significance was calculated (*t* test) and marked accordingly. ***: *p* ≤ 0.001; **: *p* ≤ 0.01; ns: *p* > 0.05, no significance. Error bars represent the standard deviation of at least 10 cells.

To investigate the effect of D_2_O concentration and incubation time on deuterium labeling of cancer cells—as well as to optimize the deuterium labeling procedure—we calculated the metabolic index from the SCRS and plotted the index as shown in Figure 3. The metabolic index is an indicator of cellular metabolic activity and is defined as the ratio of the integrated spectral intensity of the C–D (2040–2300 cm^−1^) band over the sum of the C–D and C–H bands (2800–3100 cm^−1^).

Figure 3A,C show that the metabolic index increased with increasing D_2_O concentration (from 5%–30%). This was consistent with findings from bacterial deuterium labeling, whereby a higher deuterium composition leads to a higher proportion of deuterium in proteins and lipids [16]. Nevertheless, 40% D_2_O had no benefit over 30% in terms of increasing the incorporation of deuterium into the cells (*p* values > 0.05). Thus, 30% D_2_O was chosen as the optimal concentration for deuterium labelling.

An overall ascending trend of metabolic index was demonstrated for both HCC827 and MCF-7 cells during incubation (Figure 3B,D). Here, the 12 h incubation in D_2_O was sufficient for the cells to incorporate deuterium and form C–D bonds that could be readily detected with Raman spectroscopy. This implies that the metabolic activity detection of cancer cells was independent of cell growth for the doubling times of HCC827 and MCF-7 were reported to exceed 24 h [25,26]. The metabolic index differences were significant when we increased the labeling time from 12 h to 24 h. In contrast, the difference in metabolic index is not significant in treatments with an incubation time between 24 and 36 h (*p* values > 0.05). Thus, 24 h was chosen as the optimal labeling time in our protocol.

### 3.3. Effect of Deuterium on Cell Viability

**Figure 4:**
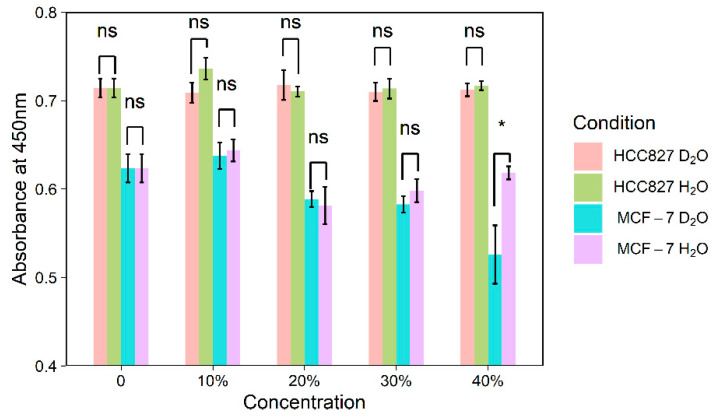
Relative cell viability after 24 h of incubation in D_2_O and H_2_O. Error bars represent the standard deviation of eight replicates. *: *p* ≤ 0.05; ns: *p* > 0.05, no significance.

To exclude the influence of D_2_O on cell viability, a series of experiments were carried out by evaluating the cell viability after D_2_O incubation using CCK-8 kit and by examining the cell morphologies via microscopy. Figure 4 shows that the absorbance at 450 nm was similar at various concentrations of D_2_O or H_2_O for 24 h except for D_2_O values over 30% (*t* test, *p* < 0.05, Table S2). The CCK-8 kit allows sensitive colorimetric assays for the determination of cell viability in cytotoxicity assays. This method detects the amount of yellow formazan dye reduced from a water-soluble tetrazolium salt by dehydrogenase activities in viable cells. The amount of the formazan dye is directly proportional to the number of living cells [27]. The results indicate that the differences between D_2_O and H_2_O on cell viability were not significant, especially when the D_2_O concentration was less than 30% (Figure 4).

Figures S4 and S5 show that the morphologies of MCF-7 were maintained after being cultured in D_2_O-containing medium. With respect to HCC827 cells, it is noted that D_2_O concentrations of up to 40% did not interfere with cell viability. In contrast, the same concentration resulted in a significant decline in cell viability of MCF-7 cells. This result justified our selection of 30% D_2_O as the optimal labeling concentration. These results of the present study are consistent with previous studies which suggested that higher intracellular D_2_O concentration may have a negative effect on cell viability [28,29]. The different growth rate of HCC827 and MCF-7 might attribute to the occurring of differences in cytotoxicity under 40% D_2_O. MCF-7 cells grow faster than HCC827 with the doubling time is 24 h versus 71 h [25,26]. The faster growing MCF-7 cells takes in more deuterium than HCC827 cells during a same incubating time, which inhibits the cell growth and reduce cell viability.

### 3.4. Chemotherapy Drug Efficacy Sensing by Single-Cell Raman-DIP

**Figure 5:**
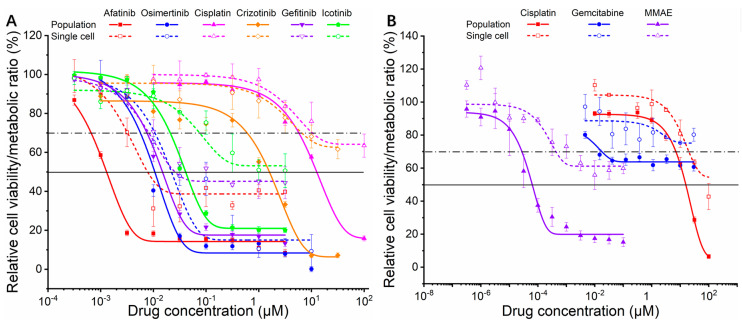
Dose–effect curve of relative cell viability obtained at the bulk level and metabolic ratio obtained at the single-cell level for HCC827 (**A**) and MCF-7 (**B**) toward different chemotherapy drugs. Colored solid curves represent the dose–effect curves at the population level. Colored dashed curves represent the dose-effect curves at the single-cell level. The horizontal solid lines and dotted-dashed lines indicate the level of 50% of relative cell viability and 70% of metabolic ratio, respectively. Error bars represent the standard deviation among the replicates.

To evaluate the reliability of our single-cell Raman-DIP approach for anticancer drug efficacy screening, we studied the metabolic index and cell viability of the two cell lines (HCC827 and MCF-7) exposed to various anticancer drugs via Raman-DIP and classical CTG assay, respectively. The CTG kit allows for sensitive luminescence assays for the determination of cell viability in cytotoxicity assays. It detects the luminescence generated by the luciferase-catalyzed transformation of luciferin that only occurs in viable cells. The luminescent signal is proportional to the number of living cells [27]. The relative cell viability was used to assess D_2_O inhibition and was defined as the percentage of luminescence of D_2_O-incubated sample to that of the negative control. The metabolic ratio was used to assess the drug inhibition detected by single-cell Raman-DIP and was defined as the percentage of metabolic index of drug-treated sample to that of the negative control sample. Figure 5A,B show the relative cell activity and metabolic ratio of HCC827 and MCF-7, respectively. The dose–effect curve clearly indicated the trend of sigmoidal fitting–the relative cell viability decreased after 72–144 h of exposure to drugs as the dose of the drug increased.

The changes in metabolic ratio by Raman-DIP after 24 h of drug exposure showed a very similar trend and was also described by sigmoidal fitting. However, the metabolic ratios of single cells were generally higher than the relative cell viabilities of cell populations (as shown by the dash lines appearing above the solid lines in Figure 5). The reason might be that the single cell metabolism indicator was measured regardless of cell numbers while effective anticancer drugs could increase the cell doubling time and lessen the total cell number [30]. Future studies based on Raman-DIP could consider both cell numbers and single cell metabolic activities to establish an even more sensitive model.

The IC_50_s of chemicals were classical benchmark to evaluate drug’s efficacy. The IC_50_s from the CTG assay were estimated with sigmoidal fitting and are presented in Table 1. The data show that HCC827 was resistant to cisplatin, while MCF-7 was resistant to cisplatin and gemcitabine (IC_50_s > 10 μM). We calculated the IC_20_s, IC_30_s, and IC_40_s from the Raman-DIP assay (listed in Table 1 and Table S3) and proposed to use IC_30_ as a criterion in the Raman-DIP approach to evaluate drug efficacy because the IC_30_ values were all on the same order of magnitude as IC_50_ from the population-level approach. These metrics give the highest sensitivity, specificity, and accuracy (100%) among all criteria (Table 1 and Table S4).

The IC_30_ results indicate that both HCC827 and MCF-7 were resistant to cisplatin, while MCF-7 was insensitive to gemcitabine. This agreed well with the results from populational analysis and other studies that use the MTT assay [27,31,32,33,34]. The CTG kit used here is a derivative of the MTT assay—a gold-standard approach for in vitro drug efficacy identification [35]. In short, our results from a panel of eight drugs on two cancer cell lines demonstrated that single-cell Raman-DIP could track the variation of cellular metabolic activity and could also shorten a lengthy 72–144 h in vitro drug sensitivity test procedure to 48 h. Hence, this reduction in time potentially lowers the cost for drug screening and speeds up the process for determining the correct cancer treatment for individuals.

### 3.5. The Heterogenous Response of Cells to Chemotherapy Drugs

Cancers are composed of mixed cell populations with diverse characteristics. Intratumor heterogeneity describes the tumor heterogeneity observed among tumor cells within one host organ [36,37] and is often associated with the heterogeneous resistance of cancer cells to anticancer drugs and treatment failure [38]. Though in vitro drug sensitivity tests, such as MTT and CTG assays on acquired tumor cells from patients could predict the outcome of the treatment and guide the medication, they are unreliable because these assays cannot elucidate the heterogeneous resistance occurred at the single-cell level. In contrast, the variation in cell heterogeneity can be detected by single-cell Raman-DIP sensing that measures metabolic activity at the single-cell level. 

**Figure 6:**
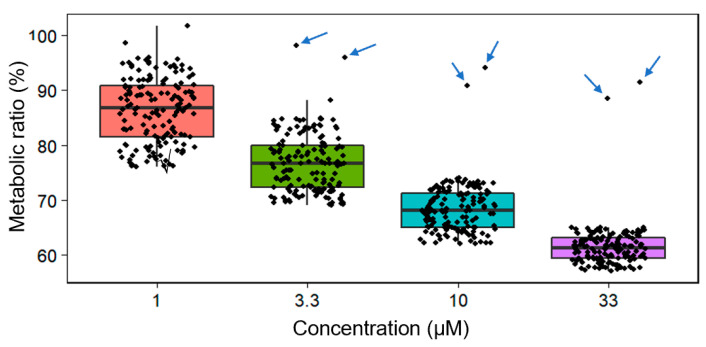
The metabolic ratio distribution of single HCC827 cells treated with crizotinib at different concentrations. Single cells (*n* = 150) were analyzed in each treatment. Some cells exhibited extreme metabolic ratios that were numerically distant from the rest of the data; these are marked with arrows.

Figure 6 exhibits a metabolic ratio of 150 single HCC827 cells exposed to crizotinib at various concentrations. Although the general pattern of descending metabolic ratio was observed with increasing drug dose, the distribution of metabolic ratio among cells from the same treatment clearly shows a subpopulation consisting of only a few (~1%) single cells with an undisturbed metabolic activity. Strong C–D bands were present in the SCRS of cells in this subpopulation. This was probably due to drug resistance. Lung cancer is highly heterogeneous with respect to metabolic activity at the single-cell level [39,40]. Our results implied the possibility of applying Raman-DIP to patient tumor cells, and in vitro assessments of the heterogeneous phenotypic drug susceptibility can thus be performed. This is a promising complement to single-cell RNA sequencing, which remains an expensive but powerful tool for genotypic heterogeneity. Both approaches can shed light on the variations in tumor heterogeneity [40,41].

## 4. Conclusions

In this study, we developed an accurate, sensitive, and rapid in vitro anticancer drug effect sensing method, i.e., Raman-DIP. The method is based on single-cell Raman microscopy coupled with deuterium labeling. Raman-DIP sensing measures the cell metabolic activity, as indicated by the C–D band in SCRS. We applied Raman-DIP to two tumor cell lines and assessed their susceptibility to eight anticancer drugs. The results showed that Raman-DIP could detect cancer cell metabolic activity at the single-cell level. The optimal labeling condition was 24 h of incubation in 30% deuterated medium. The inhibition of HCC827 and MCF-7 metabolic activity by anticancer drugs was sensitively detected via Raman-DIP. The results are consistent with the cell viability result measured by a classical MTT assay at the population level.

The features of the single-cell Raman-DIP-based method with other in vitro techniques are compared in Table 2. Raman-DIP shortened the duration of in vitro drug tests from 72–144 h to 48 h. Here, 12 h of labeling in D_2_O was sufficient for the detection of cancer cell metabolic activity at single cell level, and the assay duration could thus potentially be further reduced. Moreover, this approach solves issues with false positive results caused by toxic compounds used in other in vitro assays; hence, there was increased accuracy of drug screening. The single-cell resolution provided by Raman-DIP for identifying heterogeneous resistance was another benefit over population level assay. In conclusion, this proof-of-concept study demonstrated the potential of Raman-DIP as a reliable and novel tool to lower the attrition rate of cancer drug development and to increase patient welfare.

## Figures and Tables

**Table 1:** The IC_50_ and IC_30_ (μM) of different chemotherapy drugs and their drug resistance.

Cell Lines	Drugs	Population Level Analysis		Single-Cell Raman-DIP	
		IC_50_ (μM)	Drug Resistance	IC_30_ (μM)	Drug Resistance
HCC827	Afatinib	0.0013	S	0.0032	S
	Cisplatin	12.56	R	10.35	R
	Crizotinib	1.59	S	6.54	S
	Gefitinib	0.015	S	0.0082	S
	Icotinib	0.041	S	0.076	S
	Osimertinib	0.011	S	0.010	S
MCF-7	Cisplatin	15.67	R	20.10	R
	Gemcitabine	>30	R	>30	R
	MMAE	6.33 × 10^−5^	S	4.29 × 10^−4^	S
Sensitivity of IC_30_				1.0	
Specificity of IC_30_				1.0	
Accuracy of IC_30_				1.0	

MMAE: Monomethyl auristatin E; R: Resistant, S: Sensitive.

**Table 2:** Comparison of some in vitro anticancer drug efficacy screening techniques.

	MTT Assays	3D Cell Culture	Raman SERS	Single-Cell Raman DIP
Toxic reagent (False-positive results)	Yes	No	No	No
Culture apparatus	Multi-well plate/slide	Specially designed device	SERS chip	Multi-well plate/slide
Sensing device	Plate reader	Confocal microscope	Raman microscope	Raman microscope
Duration	72–144 h	>72 h	24–48 h	24–48 h
Cost	Low	High	Low	Low
Universality	Universal biomarker for most cancer cells and drugs	Limited types of cancer cell grow into 3D structure under lab condition	Not universal biomarker	Universal biomarker for most cancer cells and drugs
Single-cell heterogeneity sensing	No	Yes	Not demonstrated	Yes
References	[3,4,5,6,7]	[2,3]	[10,11]	This study

## Data Availability

Not applicable.

## Supplementary Materials

The following are available online at https://www.mdpi.com/article/10.3390/bios11080286/s1, Figure S1. Detailed system schematic of the home-built confocal Raman spectrometer. Figure S2. Changes in Raman intensities for nucleic acid (781 cm^−1^), protein (1240 cm^−1^), protein and lipids (1450 cm^−1^), and carbon–deuterium bond (2170 cm^−1^) of HCC827 and MCF-7 cell lines under different D2O concentrations. Figure S3. Illustration of deuterium from D_2_O incorporation to biomolecules by active cells. Figure S4. Bright-field microscopic images (50× objective) of MCF-7 cells treated with D_2_O for 12 h. Figure S5. Bright-field microscopic images (50× objective) of MCF-7 cells treated with D_2_O for 36 h. Table S1. The specifications of the confocal Raman spectrometer. Table S2. The difference of viability of cancer cells at different D_2_O/H_2_O concentrations. Table S3. IC_20_, IC_30_, and IC_40_ of different chemotherapy drugs. Table S4. Sensitivity, specificity, and accuracy of the criterions IC_20_, IC_30_, and IC_40_.
