# Supplementary material for: In Vitro Anticancer Drug Sensitivity Sensing through Single-Cell Raman Spectroscopy
Source: Biosensors (Basel). 2021 Aug 20;11(8):286. doi: 10.3390/bios11080286 (PMC8392728; doi:10.3390/bios11080286)
Supplement: Supplementary file 1 [file biosensors-11-00286-s001.zip › biosensors-1322473-supplementary.pdf]

biosensors

Article

In Vitro Anticancer Drug Sensitivity Sensing through Single-Cell Raman Spectroscopy

Jingkai Wang [1,2], Kaicheng Lin [2], Huijie Hu [1,2], Xingwang Qie [2], Wei E. Huang [3], Zhisong Cui [4,5,*], Yan Gong [2,*] and Yizhi Song [2,*]

1 School of Biomedical Engineering (Suzhou), Division of Life Sciences and Medicine, University of Science and Technology of China, Hefei 230026, China; wangjk@sibet.ac.cn (J.W.); huhj@sibet.ac.cn (H.H.)
2 Suzhou Institute of Biomedical Engineering and Technology, Chinese Academy of Sciences, Suzhou 215163, China; linkc@sibet.ac.cn (K.L.); qiexw@sibet.ac.cn (X.Q.)
3 Department of Engineering Science, University of Oxford, Parks Road, Oxford OX1 3PJ, UK; wei.huang@eng.ox.ac.uk
4 Marine Bioresources and Environment Research Center, First Institute of Oceanography, Ministry of Natural Resources of China, Qingdao 266061, China
5 Laboratory for Marine Ecology and Environmental Science, Qingdao National Laboratory for Marine Science and Technology, Qingdao 266071, China
* Correspondence: czs@fio.org.cn (Z.C.); gongy@sibet.ac.cn (Y.G.); songyz@sibet.ac.cn (Y.S.)

Citation: Wang, J.; Lin, K.; Hu, H.; Qie, X.; Huang, W.E.; Cui, Z.; Gong, Y.; Song, Y. In Vitro Anticancer Drug Sensitivity Sensing through Single-Cell Raman Spectroscopy. *Biosensors* **2021**, *11*, 286. https://doi.org/10.3390/bios11080286

Received: 16 July 2021
Accepted: 17 August 2021
Published: 20 August 2021

Publisher's Note: MDPI stays neutral with regard to jurisdictional claims in published maps and institutional affiliations.

Copyright: © 2021 by the author. Licensee MDPI, Basel, Switzerland. This article is an open access article distributed under the terms and conditions of the Creative Commons Attribution (CC BY) license (http://creativecommons.org/licenses/by/4.0/).

1. Materials and Methods

1.1. The setup of a Raman microscopy sensing system

The confocal Raman spectrometer was assembled based on an Olympus upright microscope (BX43F, Olympus, Tokyo, Japan). A detailed system schematic of the home-built confocal Raman spectrometer is shown in Figure S1. In addition, the specifications of the system are listed in Table S1.

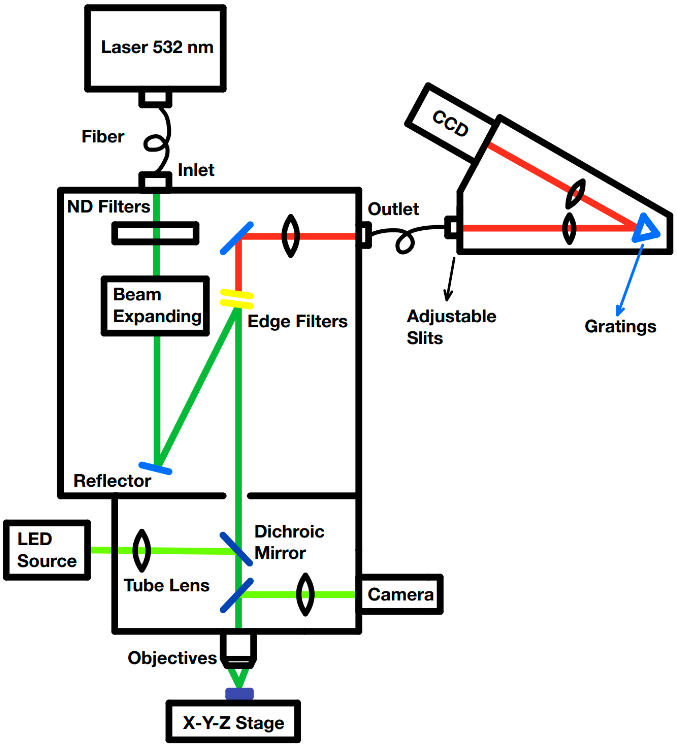

Figure S1. Detailed system schematic of the home-built confocal Raman spectrometer. ND Filters: Neutral Density Filters. The laser, confocal microscopy module, and spectrometer are connected via fibers.

Table S1. The specifications of the confocal Raman spectrometer.

Module	*Specifications*	*Value*	*Condition*
Confocal Microscopy Module	X-Y space resolution	Better than 500 nm	100× NA0.95 objective
	Z space resolution	Better than 900 nm	100× NA0.95 objective
Spectrometer Module	Spectral resolution	3 cm^{-1}	600 lines/mm grating
	Spectral range	400-3400 cm^{-1}	

1.2. Data analysis

There are several terms that are commonly used along with the description of sensitivity, specificity and accuracy. They are true positive (TP), true negative , false negative (FN), and false positive (FP) [1].

Sensitivity, specificity and accuracy are described in terms of TP, TN, FN and FP.

Sensitivity = TP/(TP + FN) = (Number of true positive assessments)/(Number of all positive assessments)

Specificity = TN/(TN + FP) = (Number of true negative assessments)/(Number of all negative assessments)

Accuracy = (TN + TP)/(TN+TP+FN+FP) = (Number of correct assessments)/Number of all assessments)

2. Results and Discussion

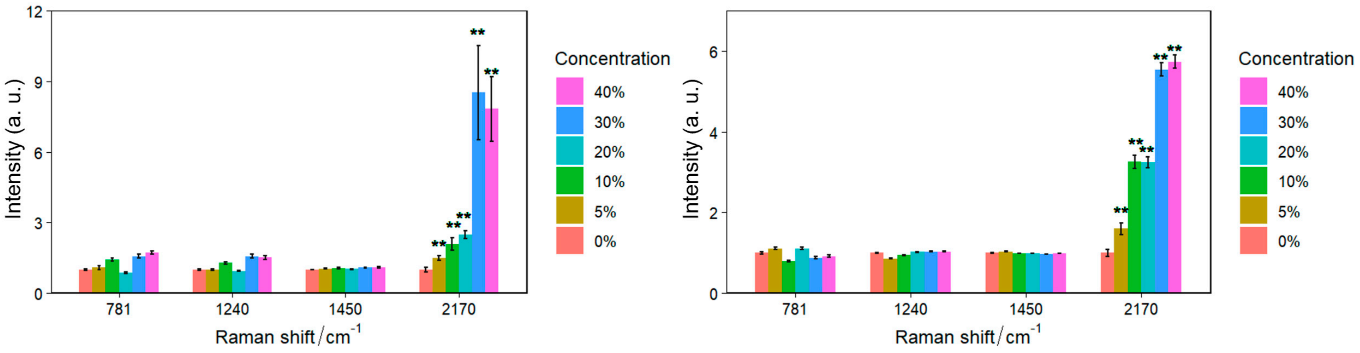

Figure S2. Changes in Raman intensities for nucleic acid (781 cm^{-1}), protein (1240 cm^{-1}), protein and lipids (1450 cm^{-1}), and carbon-deuterium bond (2170 cm^{-1}) of HCC827(A) and MCF-7 (B) cell lines under different D_2O concentrations. The intensity of all peaks in negative control (0% D_2O) was normalized. Bars represent the average intensity ($n \geq 100$). **: $p \leq 0.01$; intensities at other positions show no significant difference. The error bars represent the standard error.

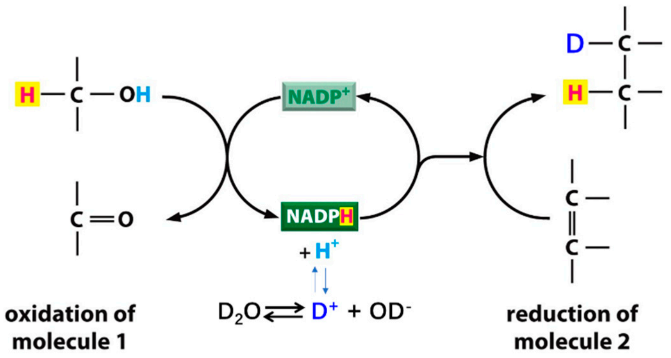

Figure S3. Illustration of deuterium from D_2O incorporation to biomolecules by active cells.

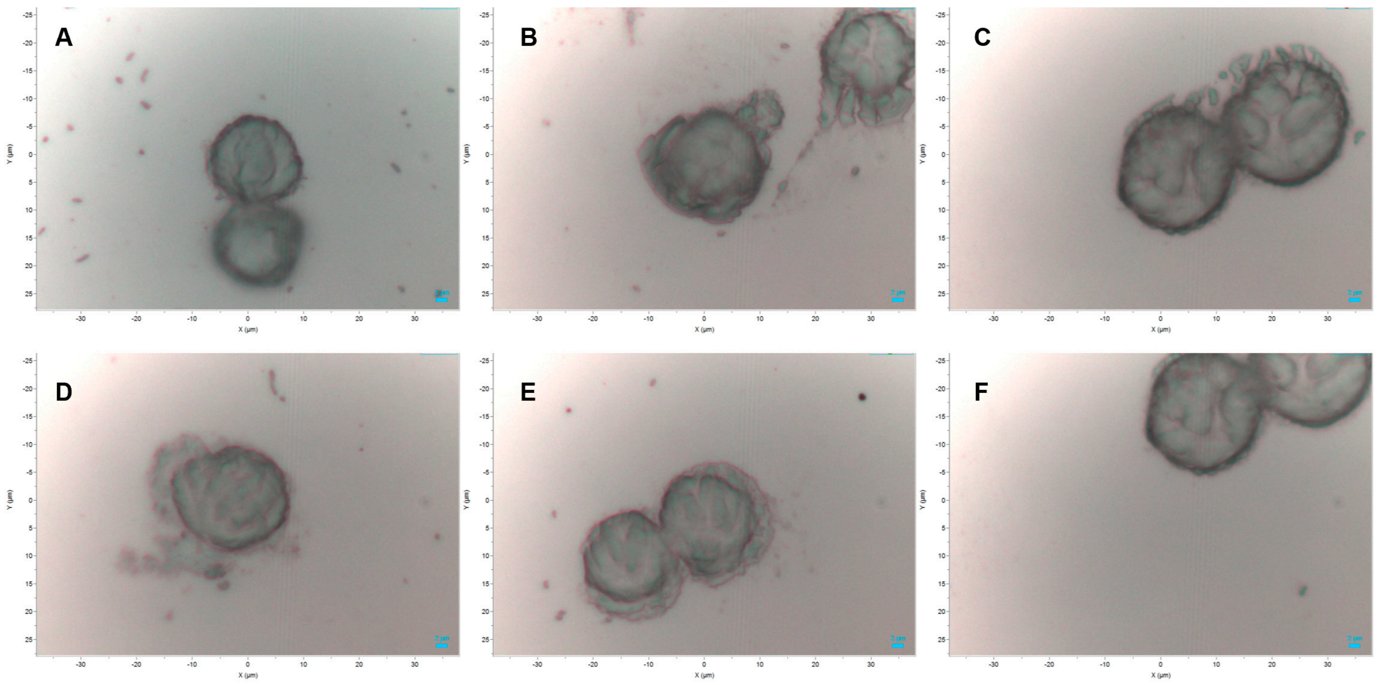

Figure S4. Bright-field microscopic images (50× objective) MCF-7 cells treated with D_2O for 12 h. Cells were fixed by 4% paraformaldehyde and transferred to aluminum slide and air-dried. The concentration of D_2O was (A) 0%, (B) 5%, (C) 10%, (D) 20%, (E) 30%, (F) 40%. The scale bars represent 2 µm.

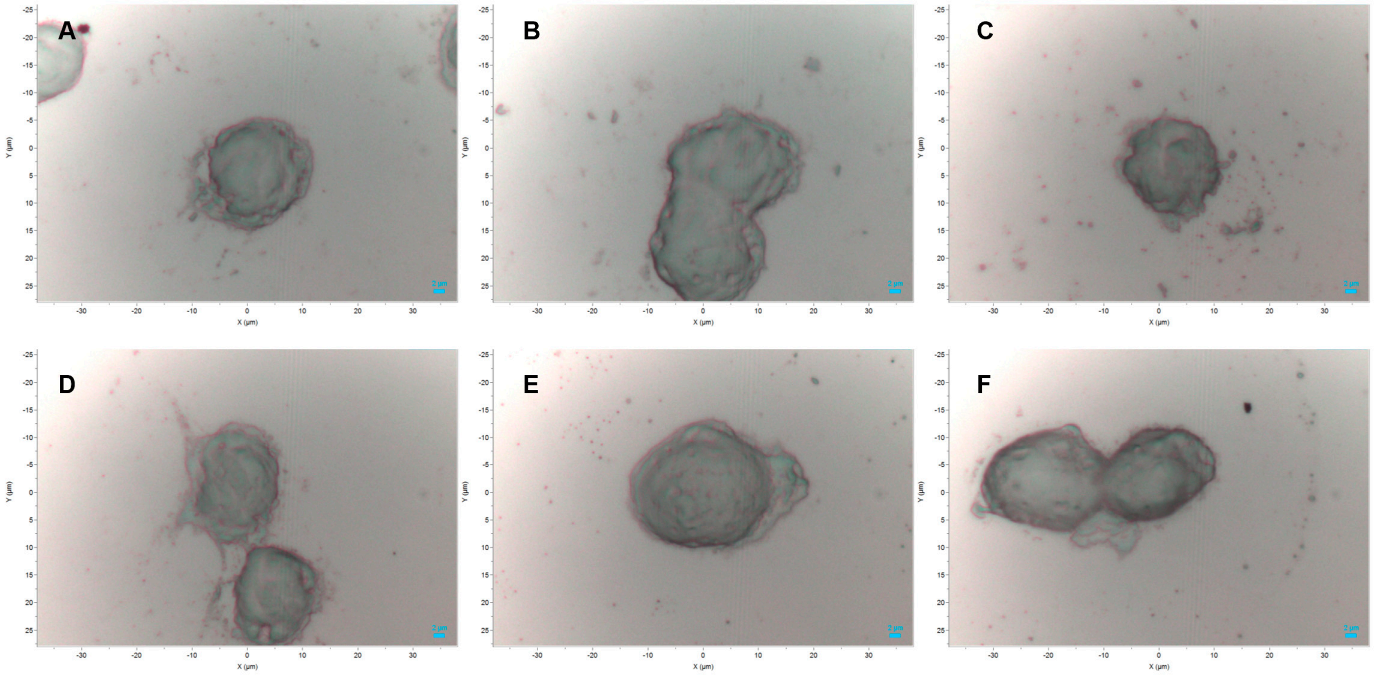

Figure S5. Bright-field microscopic images (50× objective) MCF-7 cells treated with D_2O for 36 h. Cells were fixed by 4% paraformaldehyde and transferred to aluminum slide and air-dried. The concentration of D_2O was (A) 0%, (B) 5%, (C) 10%, (D) 20%, (E) 30%, (F) 40%. The scale bars represent 2 µm.

Table S2. The difference of viability of cancer cells at different D_2O/H_2O concentrations.

Group condition	*Sample species*	*P value*	*Significance*
10% D_2O	HCC827	0.09081	No significance
	MCF-7	0.38674	No significance
20% D_2O	HCC827	0.35162	No significance
	MCF-7	0.38310	No significance
30% D_2O	HCC827	0.41138	No significance
	MCF-7	0.19432	No significance
40% D_2O	HCC827	0.31447	No significance
	MCF-7	0.02608	*

*: $p < 0.05$; No significance: $p > 0.05$

Table S3. IC_{20}, IC_{30}, IC_{40}, and IC_{50} (µM) of different chemotherapy drugs.

**Cell lines**	**Drugs**	**IC_{50} of population level analysis (µM)**	**Correlation coefficient (*r*)**	**IC_{20} of single-cell Raman-DIP (µM)**	**IC_{30} of single-cell Raman-DIP (µM)**	**IC_{40} of single-cell Raman-DIP (µM)**	**Correlation coefficient (*r*)**
	Afatinib	0.0013	0.974	0.0020	0.0032	0.0049	0.963
	Cisplatin	12.56	0.976	4.68	10.35	>100	0.994
HCC827	Crizotinib	1.59	0.997	2.82	6.54	>30	0.975
	Gefitinib	0.015	0.994	0.0046	0.0082	0.014	0.899
	Icotinib	0.041	0.995	0.034	0.076	0.16	0.995

	Osimertinib	0.011	0.995	0.0065	0.010	0.016	0.910
MCF-7	Cisplatin	15.67	0.999	11.56	20.10	37.63	0.985
	Gemcitabine	>30	0.906	2.62	>30	>30	0.803
	MMAE	6.33×10^{-5}	0.981	2.06×10^{-4}	4.29×10^{-4}	>0.1	0.854

To choose the most appropriate criterion for evaluating drug efficacy at the single-cell level, we calculated the sensitivity, specificity and accuracy of IC_{20}, IC_{30}, and IC_{40}. In the population level IC_{50} of the drugs, 10 μM serves as the benchmark for the chemotherapy drugs to evaluate their efficacy. Similar to the IC_{50}, the drug resistance results at single-cell level are summarized in Table S4. Of the three ICs, we chose IC_{30} as the final criterion for the single-cell Raman-DIP approach because it had the best sensitivity, specificity, and accuracy.

Table S4. Sensitivity, specificity, and accuracy of the criterions IC_{20}, IC_{30}, and IC_{40}.

Cell lines	Drugs	IC_{50} of population level analysis	IC_{20} of single-cell Raman-DIP	IC_{30} of single-cell Raman-DIP	IC_{40} of single-cell Raman-DIP
HCC827	Afatinib	S	S (TP)	S (TP)	S (TP)
	Cisplatin	R	S (FP)	R (TN)	R (TN)
	Crizotinib	S	S (TP)	S (TP)	R (FN)
	Gefitinib	S	S (TP)	S (TP)	S (TP)
	Icotinib	S	S (TP)	S (TP)	S (TP)
	Osimertinib	S	S (TP)	S (TP)	S (TP)
MCF-7	Cisplatin	R	R (TN)	R (TN)	R (TN)
	Gemcitabine	R	S (FP)	R (TN)	R (TN)
	MMAE	S	S (TP)	S (TP)	S (TP)
	Sensitivity		1.0	1.0	0.83
	Specificity		0.33	1.0	1.0
	Accuracy		0.78	1.0	0.89

R: Resistant, S: Sensitive. TP: true positive, TN: true negative, FN: false negative, and FP: false positive.

References

1. Zhu, W., N. Zeng, and N. Wang, *Sensitivity, Specificity, Accuracy, Associated Confidence Interval and ROC Analysis with Practical SAS ® Implementations.* NorthEast SAS users group, health care and life sciences, 2010.
